# Evaluating the 3-month post-intervention impact of a supportive text message program on mental health outcomes during the 2023 wildfires in Alberta and Nova Scotia, Canada

**DOI:** 10.3389/fpubh.2024.1452872

**Published:** 2024-12-18

**Authors:** Gloria Obuobi-Donkor, Reham Shalaby, Belinda Agyapong, Raquel da Luz Dias, Ejemai Eboreime, Lori Wozney, Vincent Israel Opoku Agyapong

**Affiliations:** ^1^Department of Psychiatry, Faculty of Medicine, Dalhousie University, Halifax, NS, Canada; ^2^Department of Psychiatry, Faculty of Medicine and Dentistry, University of Alberta, Edmonton, AB, Canada; ^3^Mental Health and Addictions Program, IWK Health, Halifax, NS, Canada

**Keywords:** wildfires, mental health, supportive text intervention, Alberta, Nova Scotia

## Abstract

**Background:**

Individuals exposed to wildfires are at risk of developing adverse mental health conditions in the months following the event. Receiving supportive text interventions during and after a wildfire event can have a significant impact on reducing mental health conditions over time.

**Objectives:**

The study aimed to assess the effectiveness of a supportive text message intervention service in reducing the severity and prevalence of psychological conditions 3 months following the 2023 wildfires in Alberta and Nova Scotia, two regions heavily affected by these natural disasters.

**Methods:**

In this longitudinal study, participants voluntarily subscribed to the Text4Hope-AB and Text4Hope-NS services, receiving supportive text interventions for 3 months. On enrolment and at 3 months post-enrolment, participants completed online surveys. The severity and prevalence of mental wellbeing, resilience, depression, anxiety, and post-traumatic stress were measured using the World Health Organization- Five Well-Being Index (WHO-5), Brief Resilience Scale (BRS), Patient Health Questionnaire 9 (PHQ-9), Generalized Anxiety Disorder - 7 scale (GAD-7), and Post-Traumatic Stress Disorder Checklist for Civilians (PCL-C) respectively. Data analysis involved using McNemar’s chi-square test and paired sample *t*-tests.

**Results:**

A total of 150 subscribers partially or fully completed both the baseline and 3-month assessments. The results show a statistically significant change in the mean scores on the WHO-5 Wellbeing Index (+ 24.6%), PHQ-9 (−17.0%), GAD-7 scale (−17.6%), PCL-C (−6.0%), and BRS (+3.2%) from baseline to 3 months. Similarly, there was a reduction, although not statistically significant, in the prevalence of low resilience (55.1 vs. 53.4%), poor mental well-being (71.6 vs. 48.3%), likely MDD (71.4 vs. 40.7%), likely GAD (42.1 vs. 33.3%), and likely PTSD (42.0 vs. 38.4%).

**Conclusion:**

The study’s findings underscore the potential of the supportive text intervention program in effectively aiding individuals who have endured natural disasters such as wildfires. Providing supportive text messages during wildfire events is a promising strategy for mitigating mental health conditions over time.

## Introduction

In recent years, mental health challenges arising from natural disasters, such as wildfires, have garnered increased attention due to their profound and enduring impacts on affected individuals and communities (1, 2). Wildfires happening naturally, especially in determined seasons, exacerbated by climate change and human activities, not only pose immediate threats to physical safety and property but also inflict significant psychological distress on those exposed to them (3, 4). The 2023 wildfire season in Canada was not exceptional; millions of hectares of vegetation were burnt (5–7), and residents had to evacuate their homes (8). The severity of the wildfire necessitated Alberta province to declare a state of emergency (8), and Nova Scotia experienced the most devastating wildfire on record (6). The combined severity of the wildfires in 2023 led to it being declared the worst in Canada’s history, surpassing all fire seasons since 2014 (9). Understanding the intricate relationship between wildfire exposure and its impact on mental well-being is crucial for efficiently devising and delivering robust interventions and support systems. This understanding is pivotal in crafting effective strategies to alleviate the enduring effects of these occurrences.

One-third of individuals exposed to wildfires experience a myriad of mental health challenges, including heightened measures of low resilience, anxiety, depression, and post-traumatic stress disorder (PTSD) (10–12). Literature has recorded a high prevalence of psychological conditions following natural disasters, with 5.8 to 54% of adult victims reporting depressive symptoms, which may last for 10 years following wildfires (9, 13, 14). Similarly, a one-month prevalence recorded in a study conducted in Fort McMurray shows 24.8, 18.0, and 13.6% in depression, anxiety, and PTSD, respectively (14). Survivors of the wildfire in Victoria exhibited increased rates of psychological incidents 3 to 4 years later (15). Psychological symptoms remained consistent throughout follow-up periods among the high-impact group (12, 15).

Regarding the escalating mental health crisis following wildfires, studies have increasingly focused on evaluating the efficacy of interventions and examining the longitudinal trajectories of mental health outcomes in affected populations (16). Given the intricate nature of mental health, understanding the evolution of mental health status over time is key for tailoring interventions to the particular needs of wildfire-affected individuals (1).

Supportive interventions, such as text message-based interventions, have shown promise in mitigating mental health symptoms and promoting resilience among disaster-affected populations (1, 4). Text-based interventions provide convenient and accessible support, allowing individuals to seek help at their own pace and convenience while reaching a more significant number of individuals simultaneously, making them particularly effective for supporting communities affected by natural disasters. The effectiveness of text-based interventions in post-disaster contexts has been demonstrated in several studies (17, 18). For example, Ruzek et al. (17) found that text-based interventions reduced symptoms of post-traumatic stress disorder (PTSD) and related issues after war and disasters (17). By providing timely and accessible support, these interventions can help people cope with the psychological challenges associated with wildfire exposure, reduce hospitalization, and facilitate recovery (19, 20).

Supportive text message intervention improves one’s resilience during a disaster (21). Resilience, a key outcome measure in this study, is conceptualized as the ability to adapt and bounce back in the face of adversity (22, 23). This involves the ability to maintain mental and emotional well-being amidst significant stress and trauma (21, 24). The study examines changes in resilience over time as a measure of the effectiveness of the Text4Hope intervention in promoting psychological well-being and coping strategies among wildfire-affected populations.

The findings will contribute to developing evidence-based strategies focusing on psychological conditions. The objective of this study is to assess the longitudinal changes in mental health status among individuals affected by wildfires over 3 months and to examine the impacts of a texting mental health service (Text4Hope-AB and Text4Hope-NS) on the severity and prevalence of psychological conditions 3 months following the wildfires.

## Methods

### Study design and setting

The study employed a longitudinal study design centered on subscribers of the Text4Hope service. It was conducted across two Canadian provinces, Alberta and Nova Scotia, which encountered wildfire. Alberta is Canada’s fourth largest province, occupying 661,848 square kilometers, while Nova Scotia covers 55,284 square kilometers. The wildfire in Alberta started late April 2023 to early November 2023, with Alberta recording approximately 2.2 million hectares burned between March 1 and October 31, 2023 (7). On May 27, 2023, Nova Scotia experienced its largest recorded wildfire in history (6). Alberta and Nova Scotia were selected as research locations because of collaboration with the regional health authorities to implement the Text4Hope program as an emergency psychological response during the wildfire season.

### Institutional review board approval

The study adhered to the guidelines in the Declaration of Helsinki and obtained approval from the Alberta Health Research Ethics Committee (Pro00086163) and the Research Ethics Board at Nova Scotia Health (REB file #1028254). Participants’ consent was obtained upon completing the online survey and submitting responses.

### Program intervention

The supportive text messages were crafted by Cognitive behavioral therapists, psychiatrists, and psychologists in collaboration with a diverse group of end users. These messages were uploaded onto a web-based platform, which automatically sent the messages daily at 9 am Mountain Time. Participants self-subscribed to the service by texting the word “HopeNS” (Nova Scotia) or HopeAB” (Alberta) to a short code and received unidirectional supportive SMS text messages for 3 months. Participants could also opt out by texting ‘STOP’ to the same shortcode. These daily supportive messages are based on cognitive behavioral therapy principles and are delivered to subscribers’ mobile phones. The messages focus on promoting resilience, coping strategies, and overall well-being. The initial message welcomed subscribers to the service. It encouraged them to voluntarily fill out a web-based baseline survey aimed at gathering demographic and clinical information primarily related to resilience, well-being, anxiety, depression, and PTSD. Participants received another text message link 6 weeks and 3 months later, inviting them to complete a follow-up web-based survey. The survey required a maximum of 10 min to complete, and participants were not incentivized to complete the surveys. Text4Hope was implemented in previous disaster contexts, including the COVID-19 pandemic (25). It is a scalable and cost-effective intervention that can reach many individuals simultaneously, making it well-suited for supporting communities affected by natural disasters. An example of the message is:

“*No matter what setbacks you have faced or challenges that lie ahead, you can succeed if you have inner strength and stay focused. Have faith in yourself, and success will be yours no matter what problems the wildfire throws at you.”* (1).

### Enrollment, inclusion/exclusion criteria

Participants self-subscribed to the program through various channels, including social media, community outreach, and referrals from mental health service providers. Eligibility criteria for participation included:

1. Age 16 years or older.
2. Residing in Alberta or Nova Scotia during the 2023 wildfires.
3. Ability to read and understand English.
4. Access to a mobile phone with text messaging capabilities.

Participant will be ineligible if they do not meet the inclusion criteria.

### Data collection

An internet-based questionnaire was utilized in data collection and powered by Research Electronic Data Capture (REDCap 13.7.1) software (26). The study commenced in May 2023. Longitudinal data collection occurred between August 2023 and December 2023, representing 3 months post-enrollment into the program and receipt of supportive text message intervention. The survey questionnaire encompassed a range of sociodemographic factors, including gender, age, ethnicity, marital status, employment status, educational attainment, and housing situation. Additionally, self-reported clinical information on mental health symptoms was collected at baseline and 3 follow-ups.

### Outcome measure

The study’s primary outcome measures were the change in mean scores of the clinical scales from baseline to 3 months. The change in mean scores of well-being, resilience, anxiety, depression, and PTSD symptoms measured on the World Health Organization- 5 Wellbeing Index (WHO-5) (27), the Brief Resilience Scale (BRS) (28), Generalized Anxiety Disorder 7-item (GAD-7) (29), Patient Health Questionnaire-9 (PHQ-9) (30) and the PTSD Checklist – Civilian Version (PCL-C) (31) respectively. Secondary outcomes were the differences in prevalence between baseline and 3 months of mental well-being, resilience, likely MDD, GAD, and PTSD.

The criteria for low resilience were an average BRS score of less than three (28) and a cut-off WHO-5 score below 13 as a criterion for poor mental well-being (27, 32). The presence of a score of 10 or higher on the GAD-7 and PHQ-9 indicated likely GAD (29) or MDD (30), while a PCL-C score of 44 or higher indicates likely PTSD (31). These measures have been shown to have strong reliability and validity (33).

### Hypothesis

Participants who subscribe to the program will have at least 20% reduced mean scores on PHQ-9, GAD-7, and PCL-C and a 20% increase in the mean scores on the BRS and WHO-5 at 3 months compared with their enrolment scores. We also hypothesized that the prevalence of poor mental well-being, low resilience, likely GAD, MDD, and PTSD will achieve a 20% lower rate at 3 months than their baseline score. The stated hypothesis is based on the findings of the literature, which recorded those participants who received supportive text intervention reduced their psychological symptoms by 20 to 50% (20, 33–38).

### Sample size considerations

To achieve a power of 80% (*β* = 0.2), a two-sided significance level of 5% (*α* < 0.05), and detecting an effect size of 0.3 between pairs, a sample size of 90 would be needed (39).

### Statistical analysis

Data from the study were analyzed using SPSS for Windows, version 28 (IBM Corporation, Armonk, NY, USA) (40). Demographic and clinical variables were examined against participants who completed the baseline and those who completed both the baseline and follow-up survey employing the chi-square or Fisher exact test. Chi-square analysis adopted a two-tailed criterion with a significance level of less than 0.05 (*α* < 0.05). The Chi-square test was adopted to assess generalizability and determine whether there are differences in the clinical and demographic characteristics between participants who completed both the baseline and follow-up surveys and participants who completed the baseline survey. Similarly, to examine the differences in the mean scores of the clinical scales at baseline and 3 months, McNemar’s chi-square test and a paired sample 2-tailed *t*-test were performed. Paired sample *t*-tests were used to assess changes in continuous variables between baseline and follow-up, while McNemar’s chi-square test was employed for categorical data. Before applying the paired sample *t*-tests, the normality assumption was assessed using the Shapiro–Wilk test, suitable for smaller sample sizes. We also conducted Levene’s test to check for homogeneity of variances. In addition, we examined the prevalence of poor mental health, low resilience, likely GAD, MDD, and PTSD at each time point, and the results were summarized as numbers and percentages. Participants with missing responses at a 3-month had their missing data imputed, employing the last observations carried forward, precisely their responses at the 6-week responses (41).

## Results

**Figure 1:**
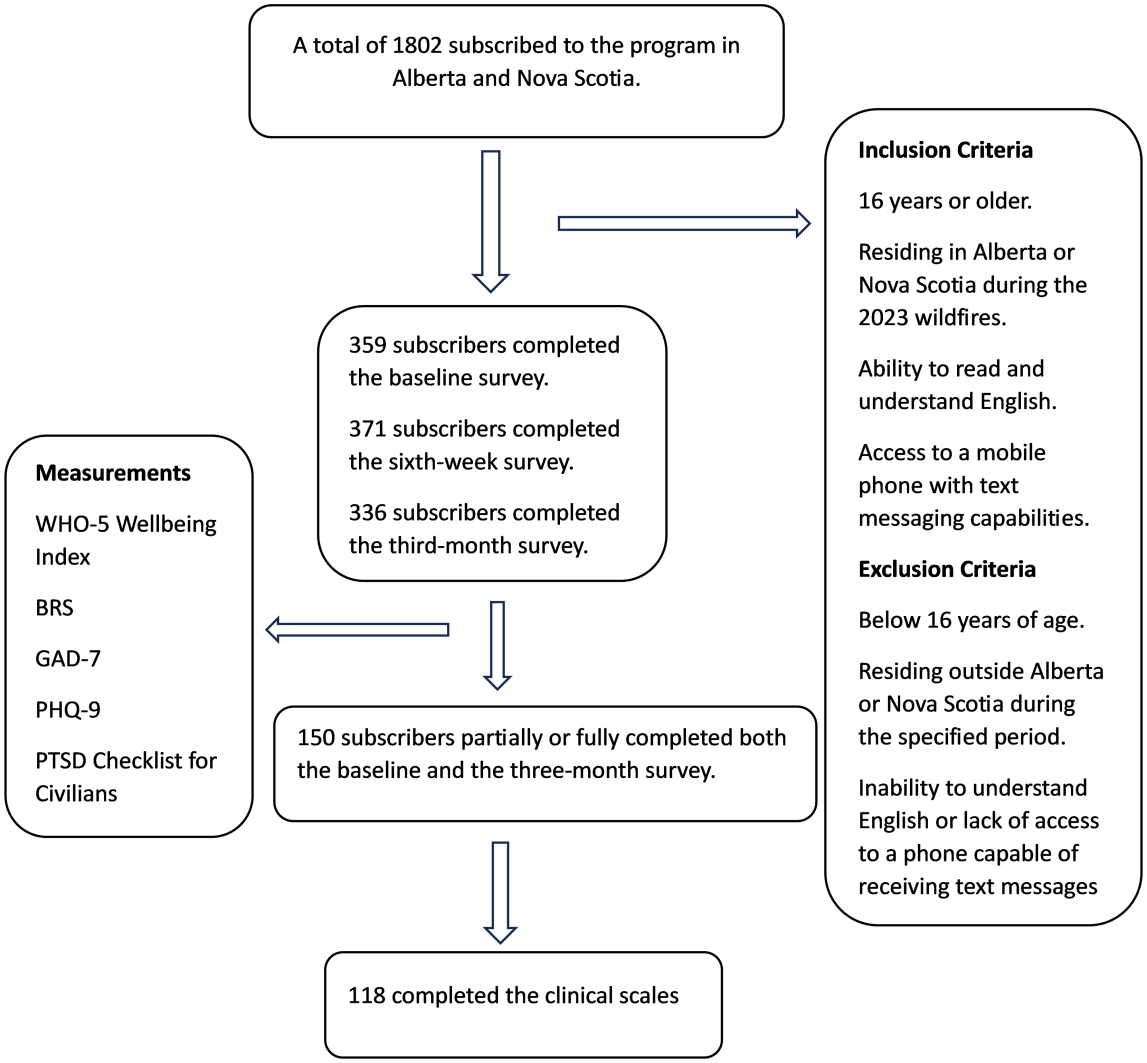
Study flowchart.

Figure 1 illustrates the flow chart of study participants in Alberta and Nova Scotia. Three hundred fifty-nine (359) participants completed the baseline survey, 371 completed the 6-week survey, and 336 completed the 3-month survey. One hundred fifty (150) participants partially or fully completed both the baseline and 3-month surveys, yielding a 41.7% response rate of participants who completed the baseline assessment, and 118 participants completed the clinical scales.

Table 1 demonstrates the distribution of the baseline demographic characteristics of study participants across the survey completion status. Out of the participants who completed their demographic information, three hundred and three were from Alberta, and fifty-six were from Nova Scotia. The result shows that the majority of the participants, 144 (40.1%), were between the ages of 31 and 50 years, 303 (84.4%) were females, 301 (83.8%) were Caucasian, and 295 (82.2%) completed postsecondary education, while 195 (54.5%) were either married, partnered, cohabiting or in a common-law. In addition, 225 (62.7%) were employed, and 234 (65.2%) owned their home. The chi-square analysis shown in Table 1 reported no significant differences in the participants’ demographic characteristics and clinical characteristics between those who completed their demographic information at baseline and those who completed follow-up surveys except for their age and employment status. Employed participants may have varying levels of availability or motivation to complete surveys. Older participants, particularly those aged 31–50, reported higher rates of completing baseline and follow-up surveys.

**Table 1:** Demographic characteristics of study participants.

Variable, *N* (%)	Survey completed		Total	Chi^2^/ Fisher’s Exact^a^	*p*-value
	Baseline only, (*N* = 356)	Baseline and follow-up (*N* = 150)			
Province					
Alberta	174 (83.3)	129 (86.0)	303 (84.4)	0.50	0.56
Nova Scotia	35 (16.7)	21 (14.0)	56 (15.6)		
Age (years)					
≤ 30	33 (15.8)	13 (8.7)	46 (12.8)	39.0	<0.001
31–50	106 (50.7)	38 (25.3)	144 (40.1)		
51–65	58 (27.8)	73 (48.7)	131 (36.5)		
> 65	12 (5.7)	26 (17.3)	38 (10.6)		
Gender					
Male	27 (12.9)	22 (14.7)	49 (13.6)	2.2 ^a^	0.35
Female	176 (84.2)	127 (84.7)	303 (84.4)		
Other	6 (2.9)	1 (0.7)	7 (1.9)		
Ethnicity					
Caucasian	176 (84.2)	125 (83.3)	301 (83.8)	3.5 ^a^	0.49
Indigenous	9 (4.3)	11 (7.3)	20 (5.6)		
Black	5 (2.4)	4 (2.7)	9 (2.5)		
Asian	9 (4.3)	7 (4.7)	16 (4.5)		
Other	10 (4.8)	3 (2.0)	13 (3.6)		
Education					
Less than high school	6 (2.9)	1 (0.7)	7 (1.9)	2.75 ^a^	0.23
High school	30 (14.4)	27 (18.0)	57 (15.9)		
Post-secondary (College, trade school, University, or Postgraduate study)	173 (82.8)	122 (81.3)	295 (82.2)		
Relationship status					
Married/Partnered/Common-Law/Cohabiting	116 (55.5)	79 (53.0)	195 (54.5)	5.39 ^a^	0.25
Single	60 (28.7)	36 (24.2)	96 (26.8)		
Separated or Divorced	25 (12.0)	21 (14.1)	46 (12.8)		
Widowed	4 (1.9)	9 (6.0)	13 (3.6)		
Other	4 (1.9)	4 (2.7)	8 (2.2)		
Employment status					
Student	12 (5.7)	3 (2.0)	15 (4.2)	14.85	0.002
Employed	142 (67.9)	83 (55.3)	225 (62.7)		
Unemployed	32 (15.3)	28 (18.7)	60 (16.7)		
Retired	23 (11.0)	36 (24.0)	59 (16.4)		
Housing status					
Own home	130 (62.2)	104 (69.3)	234 (65.2)	2.0	0.38
Renting accommodation	53 (25.4)	30 (20.0)	83 (23.1)		
Live with family and friends	26 (12.4)	16 (10.7)	42 (11.7)		
BRS					
Normal to high resilience	90 (48.9)	58 (45.0)	148 (47.3)	0.48	0.57
Low resilience	94 (51.1)	71 (55.0)	165 (52.7)		
WHO-5					
Good mental wellbeing	61 (33.5)	37 (28.9)	98 (31.6)	0.74	0.46
Poor mental wellbeing	121 (66.5)	91 (71.1)	212 (68.4)		
PHQ-9					
At most mild depression	82 (45.6)	59 (46.8)	141 (46.1)	0.05	0.91
Moderate to severe depression	98 (54.4)	67 (53.2)	165 (53.9)		
GAD-7					
At most mild anxiety	97 (54.5)	74 (59.2)	171 (56.4)	0.66	0.48
Moderate to severe anxiety	81 (45.5)	51 (40.8)	132 (43.6)		
PCL-C					
Unlikely PTSD	100 (57.1)	75 (60.5)	175 (58.5)	0.33	0.63
Likely PTSD	75 (42.9)	49 (39.5)	124 (41.5)		

^aFisher’s Exact was used.^

Among the study participants, more than half experienced low resilience (165, 52.7%), poor mental wellbeing (212, 68.4%), moderate to severe depression (165, 53.9%), and moderate to severe anxiety (132, 43.6%). Fewer reported likely PTSD symptoms (124, 41.5%).

Table 2 shows the mean scores for the BRS, WHO-5, PHQ-9, GAD-7, and PCL-C scores between participants’ baseline and 3-month time points. McNemar’s chi-square test and a paired sample 2-tailed *t*-test were employed to achieve these results. Significant improvements were found for all measures (*p* < 0.001), indicating changes in mental health status over the 3 months, with percentage changes ranging from 3.2 to 24.6%. Table 3 showed that scores on the WHO recorded the highest change from baseline with a medium effect size (Hedges’ *g* = 0.5).

**Table 2:** Changes in mental health measures from baseline to 3 months.

Measure	*N*	Mean Scores		Mean difference (95% CI)	% Change from baseline	*p* value	*t*-test	Effect size (Hedges’ *g*)
		Baseline (SD)	3 months (SD)					
BRS total score	115	2.75 (0.84)	2.84 (0.81)	−0.09 (−0.19–0.03)	3.2	<0.001	−1.65	0.1
WHO-5 total score	116	9.75 (4.95)	12.15 (5.23)	−2.40 (−3.23—1.57)	24.6	<0.001	−5.73	0.5
PHQ-9 total score	115	11.44 (6.40)	9.50 (5.89)	1.94 (0.97–2.91)	17.0	<0.001	3.95	0.3
GAD-7 total score	114	9.32 (5.37)	7.68 (5.15)	1.64 (0.80–2.46)	17.6	<0.001	3.89	0.3
PCL-C total score	112	42.34 (16.43)	39.79 (15.50)	2.55 (0.75–4.34)	6.0	0.006	2.81	0.2

**Table 3:** Changes in the prevalence of clinical parameters from baseline to 3 months (McNemar’s chi-square test).

Measure	Prevalence		Change from baseline %	*p* value
	Baseline	3 months		
Low resilience	65/118 (55.1)	63/118 (53.4)	3.1	0.8
Poor mental wellbeing	83/116 (71.6)	56/116 (48.3)	32.5	<0.001
Likely MDD	64/115 (55.7)	55/115 (47.8)	14.2	0.15
Likely GAD	48/114 (42.1)	38/114 (33.3)	20.9	0.08
Likely PTSD	47/112 (42.0)	43/112 (38.4)	8.6	0.5

Table 3 displays the prevalence of low resilience, poor mental well-being, likely MDD, GAD, and PTSD at baseline and 3-month follow-up time points. Overall, there was an apparent reduction in the prevalence of all the measures. However, the prevalence of poor mental well-being was the only condition that significantly decreased from baseline to the 3-month follow-up (*χ*^2^ = 24.1, *p* < 0.001). Conversely, the prevalence of low resilience, likely MDD, likely GAD, and likely PTSD did not exhibit statistically significant changes over the same period (*p* > 0.05).

## Discussion

This longitudinal study examined the effectiveness of Text4Hope among subscribers 3 months after the wildfire in Alberta and Nova Scotia. Our findings revealed that the mean scores on the PHQ-9, GAD-7, and PCL-C were reduced from baseline compared to the 3-month follow-up by 17.0, 17.6, and 6.0%, respectively, while mean scores on the WHO-5 and BRS increased by 24.6 and 3.2%, respectively. Our study findings partially met our hypothesis of at least a 20% reduction in prevalence and mean scores of the psychological symptoms. Notwithstanding, poor mental well-being recorded a statistically significant decrease in the prevalence (32.5%) from baseline to 3 months and an improvement in the mean scores by 24.6%, which exceeded our hypothesis of attaining at least a 20% reduction in psychological symptoms. Although the prevalence of low resilience, likely MDD, GAD, and PTSD, recorded a reduction, they were not statistically significant.

Although changes in the prevalence of depression, anxiety, PTSD, and low resilience were not statistically significant in our study, their mean scores were statistically significant. The reduction in the prevalence of psychological symptoms among wildfire victims aligns with findings from previous research (42–44). A study conducted in the aftermath of disasters recorded a similar decrease in the prevalence of mental health symptoms among affected individuals after receiving an intervention (42, 43, 45). This consistency across studies underscores the potential effectiveness of interventions in mitigating the psychological impact of wildfires on affected communities. The prevalence of psychological symptoms was higher at baseline compared to participants’ 3-month scores. The high prevalence of mental health conditions at baseline in this study also aligns with other studies that reported a high prevalence of PTSD, depression, and anxiety among affected individuals following wildfires (45–47). Individuals exposed to disasters may be at risk of developing adverse mental health conditions that worsen when intervention is not provided (48). However, participants were in significant distress at baseline, and a randomized control trial that evaluated the long-term impacts of those who did/did not receive an intervention would be beneficial. Various research has assessed the effects of a psychological intervention on mental health outcomes among disaster survivors (36, 37). These studies highlight the potential of targeted interventions to alleviate psychological distress and promote resilience in the aftermath of natural disasters.

Our study examined the demographic characteristics of study participants, mental health outcomes, and interventions during wildfires. Literature has identified significant differences in mental health status and the quest for treatment across different age groups and employment statuses among wildfire survivors (49). Wildfires can have profound and lasting impacts on the mental health of individuals, including those aged 31 to 50, females of Caucasian ethnicity with post-secondary education, married or in a committed relationship, employed, and homeowners. Research has shown that individuals in these demographics may encounter distinct challenges in coping with the aftermath of such disasters (14). Similar programs, such as Text4PTSI and Text4Wellbeing, have effectively improved mental well-being during traumatic conditions, as shown in this current study (38). Our study results showed that most of the participants own their homes; this may provide comfort and their willingness for participants to subscribe and receive supportive text messages (50). Moreover, the adaptability of text message interventions to individuals’ schedules and preferences is a key factor in their effectiveness. Subscribers can engage with the intervention at their own pace and in the privacy of their own homes, reducing potential barriers to participation (50).

Scalable and effective interventions to reduce psychological symptoms among wildfire survivors are crucial in emergency preparedness (51, 52) and appealing to health policymakers (37). The text intervention approach helps reach individuals who may otherwise face barriers to accessing traditional mental health services during disasters like wildfires (53). This implies that supportive text messages provided during wildfires could reduce the mean scores of psychological symptoms and the prevalence of poor mental well-being of wildfire victims. The results of our study provide valuable insights into the trajectories of mental health outcomes over 3 months, particularly with interventions targeting various facets of mental well-being. This finding agrees with the literature highlighting the efficacy of interventions to enhance mental wellness during crises (54–56). Such interventions have been shown to contribute positively to individuals’ overall mental health and quality of life, underscoring their importance in promoting psychological resilience and well-being.

In addition, a study conducted during the pandemic concluded that the text message intervention reduced the psychological burden of victims by approximately 25% during a 3-month follow-up (25). A focus group conducted 3 months after an intensive intervention showed positive experiences with text messaging to support behavior change maintenance (57). This suggests that the continued support provided through text messages can have lasting effects on individuals’ well-being. The baseline prevalence of mental health conditions in our study was higher than the 3-month prevalence, which may be due to the intervention received at baseline to reduce further psychological symptoms among participants. This consistency across studies underscores the potential effectiveness of interventions in mitigating the psychological impact of wildfires on affected communities. Furthermore, the importance of early detection and treatment of mental health conditions has been highlighted in various studies (58). Detecting and addressing mental health issues promptly can not only improve individual outcomes but also reduce the overall burden on healthcare services. Furthermore, the integration of physical and mental health care has been advocated to address the pervasive nature of mental health conditions globally (59).

However, while our study demonstrates positive changes in mental health outcomes, like mental well-being, resilience, depression, anxiety, and PTSD symptoms, other studies have reported contrasting findings. For instance, a longitudinal study found that mental health symptoms persisted or worsened over time among wildfire survivors (46), highlighting the variability in mental health trajectories following such disasters. These discrepancies may stem from differences in sample characteristics, intervention strategies, or follow-up periods across studies (60).

## Limitation

Considering the limitations of our study and the broader literature, these findings need to be interpreted cautiously. Firstly, the relatively limited number of participants who completed the baseline and 3-month follow-up surveys (150) suggests that this study may have been underpowered in detecting significant differences from baseline to 3 months post-intervention, further compounded by the low response rate, possibly influenced by the surveys’ online format, which could affect the generalizability of our study. Moreover, research indicates that surveys delivered via text messages have approximately 15% lower retention rates for follow-up assessments than paper-based surveys (61, 62). Secondly, all the validated scales were self-rated instead of formal diagnosis, which may cause response bias and inaccuracies due to the subjective nature of self-reporting. Again, the study was conducted in a non-randomized manner and did not include a control group. The absence of a control group restricts the ability to attribute observed changes directly to the intervention. Lastly, missing survey responses for outcome measures at the 3-month time point were imputed from the 6-week survey responses. Consequently, it is likely that the 6-week outcome data, particularly for those with imputed data, may not precisely reflect their clinical outcomes at the 3-month assessment, and much about the specific impacts of the wildfires on these participants are unknown Despite the study’s limitations, it adds to the growing body of research examining mental health outcomes and the experience of being impacted by wildfires, shedding light on the efficacy of interventions targeted at alleviating the psychological distress associated with wildfires.

## Conclusion

Results from the study show an improvement in the psychological symptoms of the study participants 3 months after the wildfires. This highlighted the importance of ongoing research to understand better the nuances of changes in mental health outcomes over time after wildfires and to develop tailored interventions that effectively address the various needs of individuals experiencing mental health challenges. Addressing the needs and challenges encountered by this population, text messaging interventions have the potential to offer valuable support and resources to promote mental well-being in the aftermath of wildfires. The Text4Hope-AB and Text4Hope-NS programs have the prospect of reducing individuals’ mental health burden 3 months after experiencing wildfires. Moreover, ongoing interventions play essential roles in promoting mental well-being and reducing the prevalence of adverse mental health conditions in the aftermath of wildfires. Continued research efforts are needed to refine intervention strategies, address disparities in mental health outcomes, and ultimately support the psychological well-being of individuals and communities affected by wildfires and other disasters.

## Data Availability

The raw data supporting the conclusions of this article will be made available by the authors, without undue reservation.
